# Association Between Eosinophilic Leukocyte Count and Hematoma Expansion in Acute Spontaneous Intracerebral Hemorrhage

**DOI:** 10.3389/fneur.2019.01164

**Published:** 2019-10-31

**Authors:** Qian Chen, Jinjin Liu, Haoli Xu, Wenwen He, Yanxuan Li, Lizhuo Jiao, Yilan Xiang, Chenyi Zhan, Jie Chen, Xiaoming Yang, Shengwei Huang, Yunjun Yang

**Affiliations:** ^1^Department of Radiology, The First Affiliated Hospital of Wenzhou Medical University, Wenzhou, China; ^2^Department of Radiology, Sir Run Run Shaw Hospital, Zhejiang University School of Medicine, Hangzhou, China; ^3^Zhejiang Provincial Key Laboratory of Aging and Neurological Disorder Research, Department of Neurosurgery, The First Affiliated Hospital of Wenzhou Medical University, Wenzhou, China

**Keywords:** intracerebral hemorrhage, hematoma expansion, leukocytes, eosinophils, growth definition, computed tomography

## Abstract

**Background/Objective:** Hematoma expansion (HE) predicts poor outcome and is an appealing treatment target in spontaneous intracerebral hemorrhage (ICH). Clinical evidence has shown an association of HE with peripheral white blood cells (WBC) count, but the individual contributions of leukocyte subtypes between literatures are described inconsistently. Our aim was to determine the relationship between admission absolute and differential leukocyte counts and HE by using different growth definitions.

**Methods:** We analyzed spontaneous ICH patients who underwent baseline cranial computed tomography and blood sampling within 6 h of stroke onset in our institution between September 2013 and August 2018. Hematoma volume was calculated using a semiautomated 3-dimensional reconstruction algorithm. According to commonly used absolute or relative growth definitions (>6 mL, >12.5 mL, or >33%), we defined 5 types of HE. A propensity score-matching analysis was performed to evaluate the influence of complete blood count components on HE across the various growth definitions. The receiver operating characteristic analysis assessed the predictive ability of leukocyte counts for HE.

**Results:** A total of 1,066 patients were included, of whom 11–21% met the 5 HE definitions. After propensity score-matching, except using the definition of >12.5 mL growth or its combination with >33% growth, both WBC and neutrophil count were independently associated with reduced risk of HE (odds ratio [OR] for 10^3^ cells increase; OR, 0.86–0.99; all *p* < 0.05) after adjusting confounders in multivariate analyses. However, monocyte count was correlated with increased risk of HE under the usage of >12.5 mL expansion definition only (OR, 1.43; *p* = 0.024). There was no association between lymphocyte count and HE (all *p* > 0.05). Regardless of the growth definition, admission eosinophil count was directly associated with the risk of HE (OR, 6.92–31.60; all *p* < 0.05), and was the best predictive subtype with area under the curve 0.64, sensitivity 69.5%, and specificity 58.9% at the optimal cut-off value of 45 cells/μL.

**Conclusions:** Growth definition affects the relationship of HE with leukocyte subtypes counting. Eosinophil count robustly predicts HE, and may be a surrogate when using an inflammatory marker to help select acute ICH patients with high expansion risk for hemostasis treatment in clinical trial and practice.

## Introduction

Intracerebral hemorrhage (ICH) is the most lethal stroke subtype with a mortality of about 40% at 1 month (1). Hematoma expansion (HE) occurs within a few hours after the initial bleeding (2), and it has been ascertained as a strong predictor of early neurological deterioration and poor long-term functional outcomes (3–5). Therefore, identifying ICH patients at high risk of early HE and taking hemostasis treatment are of great significance.

Although the chronic inflammation mediates ICH-induced secondary brain injury (6), acute inflammation reaction is beneficial for early benign transformation of the hematoma (7, 8). Peripheral white blood cells (WBC), is often used as a marker for central nervous system (CNS) inflammation and its role in the pathophysiology of acute ICH has been well-recognized (9–11). Preclinical studies have found that leukocytes infiltrate around the hematoma within hours following ICH, and neutrophils are the earliest inflammatory cells to invade the CNS (11, 12). Interleukin-27/lactoferrin-mediated neutrophil polarization can enhance hematoma clearance and improve neurological function in an animal model of ICH (7). In clinical studies, the relevance of leukocyte counts to poor ICH prognosis has received increasing attention, but no consensus is reached (13–16). Early hematoma growth, as a potential mechanism of this association, was also found in relation to admission WBC count (8). However, results with respect to the individual contributions of leukocyte subtypes to HE were inconsistent between literatures. Result from a study with large ICH population showed that the risk of HE was directly associated with monocyte count when HE was defined as a volume increase >6 mL or 30% (8). In another study defining HE as any degree of growth, investigators found no association between admission monocyte count and HE (17).

We hypothesized that growth definition might affect the stability of this association. In this study, we aimed to determine the relationship between absolute and differential leukocyte counts and HE across various growth definitions by using propensity score-matching method. Clarifying the connection of HE with leukocyte counts may provide reasonable evidence for linking inflammation to clinical prognosis in patients with acute ICH and develop potential therapeutic strategies on the HE.

## Materials and Methods

### Study Participants

We retrospectively evaluated spontaneous ICH patients admitted to our Neurological Emergency Room from September 2013 to August 2018. In total, 2,546 cases who underwent baseline computed tomography (CT) scan within 6 h and follow-up CT scan within 72 h of symptom onset were initially enrolled. Patients who met the following conditions were subsequently excluded from this study: (1) traumatic intracerebral hemorrhage or hemorrhagic transformation of a brain infarction (*n* = 746), (2) tumor, aneurysm or arteriovenous malformation presumed to be the potential cause of the bleeding (*n* = 95), (3) primary intraventricular hemorrhage or multiple cerebral hemorrhage (*n* = 47), (4) presence of encephalitis, pneumonia, or parasitic infection during the past week (*n* = 41), (5) surgical evacuation performed before the follow-up CT (*n* = 506), (6) usage of anticoagulants or antiplatelet drugs before intracerebral hemorrhage (*n* = 30), and (7) lack of complete blood count data on admission (*n* = 15). Our study was approved by the Medical Ethics Committee of The First Affiliated Hospital of Wenzhou Medical University. Written informed consent for each participant was waived owing to the retrospective nature of the study.

### Image Acquisition and Analysis

The baseline and follow-up CT scans were performed using a standard clinical protocol with an axial technique of 5-mm section thickness, tube current of 80 mA, and tube voltage of 120 kV(p). All CT images were acquired from the picture archiving and communication system (PACS) and saved as DICOM format for further evaluation. Two experienced radiologists who were blinded to clinical and laboratory data independently analyzed the images. Volumetric calculation of hematoma was completed through 3-dimensional reconstruction of the regions of interest obtained by manually depicting the lesions circumference in multiple successive layers. The volume extended to the ventricles did not be calculated. When the hematoma boundary at a certain layer was unclear, it was settled by joint discussion of the 2 readers. To assess the reliability of volume measurements between the 2 observers, we randomly selected 100 patients from the entire sample and analyzed the consistency by using the intra-group correlation coefficient (ICC). Hematoma location was also evaluated and divided into lobe, deep, and infratentorial. Both imaging assessments and volume measurements were conducted at the post-processing station (GE Healthcare Advantage Workstation; Version 4.6). According to the most commonly used definitions of HE in previous studies, we categorized HE as >33% growth, >6 mL growth, >12.5 mL growth, and their combinations (>6 mL or 33%, and >12.5 mL or 33%).

### Clinical and Laboratory Variables

Demographics (sex and age) and clinical data (glasgow coma scale score, history of hypertension, diabetes mellitus, smoking, alcohol consumption, coronary heart disease, cerebral infarction, and cerebral hemorrhage) were reviewed from the inpatient medical records. The time from symptom onset to baseline CT was also calculated. We collected laboratory parameters from the emergency medical record system, including glucose level, platelet count, international normalized ratio, total WBC count, and differential leukocyte counts (neutrophil count, monocyte count, lymphocyte count, and eosinophil count). Routine blood sampling for each participant was completed within 1 h after admission, but prior to the baseline CT imaging. Before testing, venous blood sample was placed in a 2.0 mL of disposable collection tube, which contained EDTA-K2 for the purpose of anticoagulation. Complete blood count was measured by an automatic XE-2100 hematology analyzer (Sysmex Corporation, Kobe, Japan).

### Statistical Analysis

All statistical analyses were performed using IBM SPSS Statistics (Version 22.0). First, the entire cohort were dichotomized according to the 2 combined definitions of HE. The discrete variables were expressed as frequency (percentage), and the continuous variables were presented as mean ± standard deviation (SD) or median [interquartile range (IQR)]. The demographic, clinical, and radiological characteristics were compared between patients with HE and those without. Statistical significance for the difference between the 2 groups was examined by χ^2^ test or the Fisher exact test for categorical variables, and by student *t*-test or Mann-Whitney *U*-test for continuous variables. Multivariable logistic regression models were performed to initially evaluate the associations of ICH expansion risk with absolute and differential leukocyte counts. The variables with *p* < 0.05 from both univariate analyses were forced into the multivariate model.

Subsequently, a propensity score-matching analysis was used to balance the distribution of covariates between patients who experienced HE and those without. A propensity score was calculated for each patient using logistic regression analysis, and the variables with *p* < 0.2 in the above univariate analyses were introduced into the propensity model. The included variables are as follows: sex, hypertension, drinking, history of cerebral hemorrhage, glasgow coma scale score, platelet count, intraventricular hemorrhage, baseline ICH volume, and time from onset to initial CT. Patients with HE were then matched 1:1 without replacement to patients who did not experience HE using the nearest neighbor approach with a 0.02 caliper width. Multivariate logistic regression model was further performed to assess the relationship of leukocyte counts with HE across the various HE definitions, adjusting for predictors of HE that were previously validated in the randomized clinical trial and multicenter prospective study (18–20). Finally, in the entire cohort, we performed a receiver operating characteristic analysis to assess the discrimination ability of different leukocyte counts components for predicting HE. A value of *p* < 0.05 (2 sided) was considered statistically significant.

## Results

A total of 1,066 patients met the inclusion criteria and were analyzed. With the 5 types of growth definition, HE occurred in 200 (18.8%), 116 (10.9%), 174 (16.3%), 225 (21.1%), 182 (17.1%) of all patients, respectively. There were 699 men and 367 women, and the mean age was 61.5 ± 12.6 years (range 25–95 years). The ICC in volumetric measurement between the 2 observers was 0.96 (95% confidence interval [CI], 0.95–0.98), indicating an excellent consistency.

In the entire cohort, univariate analyses based on the 2 combined HE definitions showed that the clinical and imaging characteristics presented similar trend between patients with HE and non-HE (Tables 1, 2). Patients with HE had a higher median baseline hematoma volume than those without (24.4 [13.9–35.4] vs. 16.2 [9.1–27.0] mL; 21.3 [12.6–30.6] vs. 17.1 [9.4–28.3] mL; *p* < 0.001 and *p* = 0.012, respectively), and they had a shorter time from symptom onset to initial CT (both *p* < 0.001). Patients who experienced HE also had a lower admission glasgow coma scale score compared to patients without HE (both *p* < 0.001). History of cerebral hemorrhage was more frequently observed among individuals with HE (*p* = 0.015; *p* = 0.003), while hypertension was less commonly in those individuals (*p* = 0.001; *p* = 0.008). Besides, patients with HE were more likely to be male than those with non-HE (76.4 vs. 62.7%; 76.9 vs. 63.2%; both *p* < 0.001). Multivariable analyses (Table 3) showed that in addition to using the definition of >12.5 mL growth, both WBC and neutrophil count were independently associated with reduced risk of HE (odds ratio [OR] for 10^3^ cells increase; OR, 0.93–0.95 and OR, 0.92–0.96, respectively; all *p* < 0.05) after adjusting for risk factors selected in the univariate analyses. However, monocyte count was correlated with increased risk of HE only when using the growth definition of >12.5 mL (OR, 1.98; *p* = 0.009). There was no significant association between lymphocyte count and HE (all *p* > 0.05). Regardless of the growth definition, the risk of HE was directly associated with eosinophil count (OR, 3.64–6.71; all *p* < 0.05).

**Table 1:** Comparison of baseline characteristics between HE (>6 mL or 33%) and non-HE groups in the original cohort.

**Characteristics**	**Non-HE (*n* = 841)**	**HE (*n* = 225)**	***P-*value**
Age	61.6 ± 12.7	60.9 ± 12.5	0.468
Male sex	527 (62.7)	172 (76.4)	<0.001*
Hypertension	680 (80.9)	160 (71.1)	0.001*
Diabetes mellitus	94 (11.2)	25 (11.1)	0.978
Current or previous smoking	235 (27.9)	66 (29.3)	0.681
Current or previous drinking	232 (27.6)	69 (30.7)	0.362
History of coronary heart disease	10 (1.2)	3 (1.3)	0.861
History of cerebral infarction	32 (3.8)	7 (3.1)	0.622
History of cerebral hemorrhage	29 (3.5)	16 (7.1)	0.015*
Glasgow coma scale score	14 (11–15)	11 (8–15)	<0.001*
International normalized ratio	0.99 (0.94–1.04)	0.99 (0.94–1.05)	0.209
Glucose level, mmol/L	7.0 (6.0–8.6)	7.0 (6.0–8.2)	0.631
Platelet count, 10^3^ cells/μL	204 (168–244)	199 (153–238)	0.06
Time from onset to initial CT, h	3.0 (2.0–4.5)	2.5 (1.5–3.5)	<0.001*
**Location of ICH**			**0.242**
Lobe	76 (9.0)	16 (7.1)	
Deep	707 (84.1)	199 (88.4)	
Infratentorial	58 (6.9)	10 (4.4)	
Baseline ICH volume, mL	16.2 (9.1–27.0)	24.4 (13.9–35.4)	<0.001*
Intraventricular extension	291 (34.6)	93 (41.3)	0.062

*Continuous variables are presented as mean ± SD or median (IQR); Categorical variables are presented as n (%)*.

*ICH, intracerebral hemorrhage; HE, hematoma expansion; CT, computed tomography; SD, standard deviation; IQR, interquartile range*.

* *Significant at P < 0.05*.

**Table 2:** Comparison of baseline characteristics between HE (>12.5 mL or 33%) and non-HE groups in the original cohort.

**Characteristics**	**Non-HE (*n* = 884)**	**HE (*n* = 182)**	***P-*value**
Age	61.6 ± 12.6	60.7 ± 12.9	0.354
Male sex	559 (63.2)	140 (76.9)	<0.001*
Hypertension	710 (80.3)	130 (71.4)	0.008*
Diabetes mellitus	98 (11.1)	21 (11.5)	0.897
Current or previous smoking	252 (28.5)	49 (26.9)	0.718
Current or previous drinking	242 (27.4)	59 (32.4)	0.169
History of coronary heart disease	12 (1.4)	1 (0.5)	0.366
History of cerebral infarction	33 (3.7)	6 (3.3)	0.775
History of cerebral hemorrhage	30 (3.4)	15 (8.3)	0.003*
Glasgow coma scale score	14 (10–15)	12 (8–15)	<0.001*
International normalized ratio	0.99 (0.94–1.04)	0.99 (0.94–1.05)	0.385
Glucose level, mmol/L	7.0 (6.1–8.6)	6.9 (6.0–8.2)	0.275
Platelet count, 10^3^ cells/μL	203 (168–243)	201 (149–239)	0.134
Time from onset to initial CT, h	3.0 (2.0–4.5)	2.5 (1.5–3.5)	<0.001*
**Location of ICH**			**0.243**
Lobe	81 (9.2)	11 (6.0)	
Deep	744 (84.2)	162 (89.0)	
Infratentorial	59 (6.7)	9 (4.9)	
Baseline ICH volume, mL	17.1 (9.4–28.3)	21.3 (12.6–30.6)	0.012*
Intraventricular extension	312 (35.3)	72 (39.6)	0.275

*ICH, intracerebral hemorrhage; HE, hematoma expansion; CT, computed tomography; SD, standard deviation; IQR, interquartile range*.

* *Significant at P < 0.05*.

**Table 3:** Multivariate analyses of predicting hematoma expansion in the original cohort.

	**Crude OR (95%CI)**	***P-*value**	**Adjusted^**a**^ OR (95%CI)**	***P-*value**
**>6 mL growth (*****N*** **=** **200)**				
WBC count	0.96 (0.92–1.01)	0.088	0.94 (0.90–0.99)	0.021
Neutrophil count	0.94 (0.90–0.99)	0.008	0.93 (0.89–0.98)	0.008
Monocyte count	1.74 (1.10–2.78)	0.019	1.45 (0.89–2.35)	0.131
Lymphocyte count	1.27 (1.09–1.49)	0.003	1.09 (0.90–1.31)	0.385
Eosinophil count	7.11 (2.30–21.97)	0.001	3.64 (1.04–12.7)	0.043
**>12.5 mL growth (*****N*** **=** **116)**				
WBC count	0.97 (0.92–1.02)	0.234	0.98 (0.92–1.04)	0.495
Neutrophil count	0.94 (0.89–0.99)	0.023	0.96 (0.91–1.02)	0.229
Monocyte count	2.09 (1.26–3.48)	0.004	1.98 (1.19–3.30)	0.009
Lymphocyte count	1.35 (1.13–1.61)	0.001	1.14 (0.92–1.40)	0.233
Eosinophil count	11.39 (3.27–39.66)	<0.001	5.03 (1.13–22.3)	0.034
**>33% growth (*****N*** **=** **174)**				
WBC count	0.92 (0.88–0.97)	0.001	0.94 (0.89–0.99)	0.02
Neutrophil count	0.89 (0.85–0.94)	<0.001	0.96 (0.87–0.97)	0.003
Monocyte count	1.79 (1.12–2.88)	0.016	1.62 (0.99–2.64)	0.056
Lymphocyte count	1.42 (1.21–1.67)	<0.001	1.18 (0.99–1.42)	0.072
Eosinophil count	19.12 (5.81–62.94)	<0.001	6.71 (1.73–25.9)	0.006
**>6 mL or 33% growth (*****N*** **=** **225)**				
WBC count	0.95 (0.91–0.99)	0.008	0.93 (0.89–0.98)	0.004
Neutrophil count	0.92 (0.88–0.96)	<0.001	0.92 (0.87–0.96)	<0.001
Monocyte count	1.73 (1.09–2.75)	0.019	1.44 (0.90–2.30)	0.127
Lymphocyte count	1.37 (1.17–1.59)	<0.001	1.19 (0.99–1.42)	0.053
Eosinophil count	10.38 (3.37–32.02)	<0.001	4.66 (1.28–17.02)	0.02
**>12.5 mL or 33% growth (*****N*** **=** **182)**				
WBC count	0.93 (0.89–0.98)	0.004	0.95 (0.90–0.99)	0.034
Neutrophil count	0.90 (0.86–0.95)	<0.001	0.93 (0.88–0.98)	0.005
Monocyte count	1.80 (1.12–2.89)	0.015	1.61 (0.92–2.61)	0.124
Lymphocyte count	1.41 (1.20–1.66)	<0.001	1.20 (0.92–1.43)	0.112
Eosinophil count	17.07 (5.25–55.49)	<0.001	6.51 (1.71–24.82)	0.006

*OR for 10^3^ cells increase. N represents the number of positive hematoma expansion*.

a *Adjusted by sex, hypertension, history of cerebral hemorrhage, glasgow coma scale score, time from symptom onset to initial CT, and baseline hematoma volume. WBC, white blood cell; OR, odds ratio; CI, confidence interval; CT, computed tomography*.

Propensity score-matching analyses resulted in 380 (190 pairs), 232 (116 pairs), 326 (163 pairs), 432 (216 pairs), and 358 (179 pairs) patients being matched across the 5 types of HE definition. In the propensity score-matched cohorts, univariate analyses demonstrated that there were no significant difference between HE and non-HE groups regarding the baseline clinical and radiological characteristics (all *p* > 0.05; Tables 4, 5). The admission absolute and differential leukocyte counts in patients with and without HE were summarized in Table 6. As shown in Table 6, eosinophil count was significantly associated with HE before and after propensity score matching (all *p* < 0.05). After adjusting for proven predictors of HE including history of ICH, glasgow coma scale score, time to initial CT, and baseline ICH volume, the results of multivariate regression model were similar to the findings before propensity score-matching analysis (Table 7). The association between WBC (OR, 95% CI; 0.96, 0.90–1.02; *p* = 0.22) and neutrophil counts (OR, 95% CI; 0.93, 0.87–1.00; *p* = 0.058) and HE were no longer significant when using the definition of >12.5 mL or 33% growth, whereas eosinophil count remained significantly associated with HE irrespective of the growth definition (OR, 6.92–31.60; all *p* < 0.05).

**Table 4:** Comparison of variable in the HE (>6 mL or 33%) and non-HE groups after propensity score-matching.

**Variable**	**Non-HE (*n* = 216)**	**HE (*n* = 216)**	***P-*value**
Age	60.8 ± 12.4	61.0 ± 12.6	0.896
Male sex	169 (78.2)	163 (75.5)	0.494
Hypertension	168 (77.8)	152 (70.4)	0.099
Diabetes mellitus	17 (7.9)	23 (10.6)	0.319
Current or previous smoking	75 (34.7)	65 (30.1)	0.304
Current or previous drinking	71 (32.9)	66 (30.6)	0.605
History of coronary heart disease	3 (1.4)	2 (0.9)	1.000
History of cerebral infarction	9 (4.2)	6 (2.8)	0.43
History of cerebral hemorrhage	12 (5.6)	13 (6.0)	0.837
Glasgow coma scale score	12 (9–14)	12 (8–14)	0.389
International normalized ratio	1.00 (0.94–1.05)	0.99 (0.94–1.05)	0.584
Glucose level, mmol/L	7.0 (6.0–8.4)	7.0 (6.0–8.2)	0.794
Platelet count, 10^3^ cells/μL	187 (154–231)	201 (155–239)	0.09
Time from onset to initial CT, h	2.5 (1.5–3.5)	2.5 (1.5–3.5)	0.477
**Location of ICH**			**0.092**
Lobe	21 (9.7)	15 (6.9)	
Deep	192 (88.9)	191 (88.4)	
Infratentorial	3 (1.4)	10 (4.6)	
Baseline ICH volume, mL	21.6 (13.8–33.3)	24.1 (13.9–34.6)	0.653
Intraventricular extension	82 (38.0)	88 (40.7)	0.555

*ICH, intracerebral hemorrhage; HE, hematoma expansion; CT, computed tomography*.

**Table 5:** Comparison of variable in the HE (>12.5 mL or 33%) and non-HE groups after propensity score-matching.

**Variable**	**Non-HE (*n* = 179)**	**HE (*n* = 179)**	***P-*value**
Age	60.6 ± 12.3	60.6 ± 12.9	0.99
Male sex	144 (80.4)	137 (76.5)	0.368
Hypertension	141 (78.8)	128 (71.5)	0.112
Diabetes mellitus	13 (7.3)	20 (11.2)	0.201
Current or previous smoking	66 (36.9)	49 (27.4)	0.07
Current or previous drinking	62 (34.6)	57 (31.8)	0.575
History of coronary heart disease	4 (2.2)	1 (0.6)	0.371
History of cerebral infarction	7 (3.9)	6 (3.4)	0.778
History of cerebral hemorrhage	30 (3.4)	15 (8.3)	0.398
Glasgow coma scale score	13 (9–15)	12 (8–15)	0.327
International normalized ratio	1.00 (0.95–1.05)	0.99 (0.94–1.05)	0.593
Glucose level, mmol/L	7.0 (5.9–8.4)	6.9 (6.0–8.2)	0.908
Platelet count, 10^3^ cells/μL	192 (158–233)	201 (149–239)	0.517
Time from onset to initial CT, h	2.0 (1.5–3.0)	2.0 (1.5–3.5)	0.383
**Location of ICH**			**0.146**
Lobe	16 (8.9)	11 (6.1)	
Deep	159 (88.8)	159 (88.8)	
Infratentorial	4 (2.2)	9 (5.0)	
Baseline ICH volume, mL	20.3 (13.1–32.1)	21.2 (12.6–30.6)	0.658
Intraventricular extension	63 (35.2)	70 (39.1)	0.443

*ICH, intracerebral hemorrhage; HE, hematoma expansion; CT, computed tomography*.

**Table 6:** Admission absolute and differential leukocyte counts before and after propensity score-matching.

	**Original cohort**			**Propensity score-matched cohort**		
**>6 mL**	**Non-HE (*****n*** **=** **866)**	**HE (*****n*** **=** **200)**	***P*****-value**	**Non-HE (*****n*** **=** **190)**	**HE (*****n*** **=** **190)**	***P*****-value**
WBC count	9,210 (7,178–12,040)	9,160 (6,530–11,188)	0.061	9,445 (7,513–12,193)	9,160 (6,530–11,188)	0.035
Neutrophil count	7,400 (4,845–10,300)	6,505 (4,100–9,088)	0.001	7,500 (5,325–10,275)	6,505 (4,100–9,088)	0.003
Monocyte count	455 (330–600)	500 (400–625)	0.02	500 (400–600)	500 (400–625)	0.306
Lymphocyte count	1,200 (850–1,700)	1,400 (925–2,023)	0.001	1,200 (900–1,700)	1,400 (925–2,023)	0.017
Eosinophil count	30 (0–100)	100 (10–160)	<0.001	25 (0–100)	100 (10–160)	0.001
**>12.5 mL**	**Non-HE (*****n*** **=** **950)**	**HE (*****n*** **=** **116)**	***P*****-value**	**Non-HE (*****n*** **=** **116)**	**HE (*****n*** **=** **116)**	***P*****-value**
WBC count	9,190 (7,155–12,000)	9,220 (6,265–11,493)	0.15	9,160 (7,468–11,165)	9,220 (6,265–11,493)	0.299
Neutrophil count	7,240 (4,828–10,200)	6,330 (3,740–8,900)	0.003	7,200 (5,300–9,825)	6,330 (3,740–8,900)	0.028
Monocyte count	460 (330–600)	500 (400–700)	0.006	425 (300–600)	500 (400–700)	0.011
Lymphocyte count	1,200 (848–1,700)	1,425 (1,015–2,275)	<0.001	1,200 (900–1,600)	1,425 (1,015–2,275)	0.014
Eosinophil count	30 (0–100)	100 (10–198)	<0.001	100 (0–150)	100 (10–198)	0.003
**>33%**	**Non-HE (*****n*** **=** **892)**	**HE (*****n*** **=** **174)**	***P*****-value**	**Non-HE (*****n*** **=** **163)**	**HE (*****n*** **=** **163)**	***P*****-value**
WBC count	9,305 (7,260–12,040)	8,715 (6,215–10,810)	0.001	9,140 (7,200–11,570)	8,715 (6,215–10,810)	0.024
Neutrophil count	7,400 (4,978–10,300)	5,655 (3,780–8,370)	<0.001	7,000 (5,000–9,600)	5,655 (3,780–8,370)	0.001
Monocyte count	460 (330–600)	500 (388–630)	0.023	480 (400–600)	500 (388–630)	0.176
Lymphocyte count	1,200 (810–1,690)	1,500 (1,068–2,200)	<0.001	1,300 (900–2,000)	1,500 (1,068–2,200)	0.031
Eosinophil count	20 (0–100)	100 (18–200)	<0.001	10 (0–100)	100 (18–200)	0.003
**>6 mL or 33%**	**Non-HE (*****n*** **=** **841)**	**HE (*****n*** **=** **225)**	***P*****-value**	**Non-HE (*****n*** **=** **216)**	**HE (*****n*** **=** **216)**	***P*****-value**
WBC count	9,300 (7,205–12,105)	8,820 (6,425–11,035)	0.004	9,045 (7,185–11,685)	8,820 (6,425–11,035)	0.07
Neutrophil count	7,400 (4,920–10,305)	6,100 (3,870–8,600)	<0.001	7,250 (5,000–9,900)	6,100 (3,870–8,600)	0.003
Monocyte count	460 (330–600)	500 (385–620)	0.031	420 (400–600)	500 (385–620)	0.109
Lymphocyte count	1,200 (840–1,645)	1,420 (975–2,100)	<0.001	1,200 (900–1,725)	1,420 (975–2,100)	0.021
Eosinophil count	30 (0–100)	100 (15–165)	<0.001	85 (0–100)	100 (15–165)	0.001
**>12.5 mL or 33%**	**Non-HE (*****n*** **=** **884)**	**HE (*****n*** **=** **182)**	***P*****-value**	**Non-HE (*****n*** **=** **179)**	**HE (*****n*** **=** **179)**	***P*****-value**
WBC count	9,300 (7,243–12,033)	8,725 (6,248–10,948)	0.001	8,890 (7,142–10,985)	8,725 (6,248–10,948)	0.188
Neutrophil count	7,400 (4,963–10,300)	5,800 (3,800–11,770)	<0.001	6,900 (4,775–8,875)	5,800 (3,800–11,770)	0.013
Monocyte count	460 (330–600)	500 (380–630)	0.026	500 (400–600)	500 (380–630)	0.075
Lymphocyte count	1,200 (810–1,690)	1,500 (1,000–2,193)	<0.001	1,200 (900–1,900)	1,500 (1,000–2,193)	0.019
Eosinophil count	20 (0–100)	100 (10–193)	<0.001	30 (0–100)	100 (10–193)	0.001

*Data are presented as median (IQR) [Unit: cells/μL]. HE, hematoma expansion; IQR, interquartile range*.

**Table 7:** Multivariate analyses of predicting hematoma expansion in the propensity score-matched cohorts.

	**Adjusted^**a**^ OR**	**95% CI**	***P*-value**
**>6 mL growth**			
WBC count	0.93	0.87–0.98	0.021
Neutrophil count	0.91	0.86–0.97	0.006
Monocyte count	1.48	0.75–2.93	0.252
Lymphocyte count	1.17	0.92–1.48	0.192
Eosinophil count	9.36	1.46–59.68	0.018
**>12.5 mL growth**			
WBC count	0.98	0.91–1.06	0.599
Neutrophil count	0.95	0.88–1.03	0.224
Monocyte count	1.43	1.04–1.97	0.024
Lymphocyte count	1.34	0.98–1.84	0.061
Eosinophil count	14.50	1.17–178.88	0.037
**>33% growth**			
WBC count	0.93	0.87–0.99	0.032
Neutrophil count	0.91	0.85–0.97	0.007
Monocyte count	1.91	0.82–4.42	0.131
Lymphocyte count	1.17	0.92–1.48	0.191
Eosinophil count	11.04	1.47–82.66	0.019
**>6 mL or 33% growth**			
WBC count	0.94	0.89–0.99	0.04
Neutrophil count	0.92	0.87–0.98	0.009
Monocyte count	1.87	0.89–3.91	0.096
Lymphocyte count	1.20	0.97–1.50	0.09
Eosinophil count	6.92	1.20–39.78	0.03
**>12.5 mL or 33% growth**			
WBC count	0.96	0.90–1.02	0.22
Neutrophil count	0.93	0.87–1.00	0.058
Monocyte count	2.03	0.87–4.71	0.1
Lymphocyte count	1.20	0.94–1.52	0.138
Eosinophil count	31.60	3.23–308.70	0.003

*OR for 10^3^ cells increase*.

a *Adjusted for history of cerebral hemorrhage, glasgow coma scale score, time to initial computed tomography, and baseline hematoma volume. WBC, white blood cell; OR, odds ratio; CI, confidence interval*.

**Figure 1:**
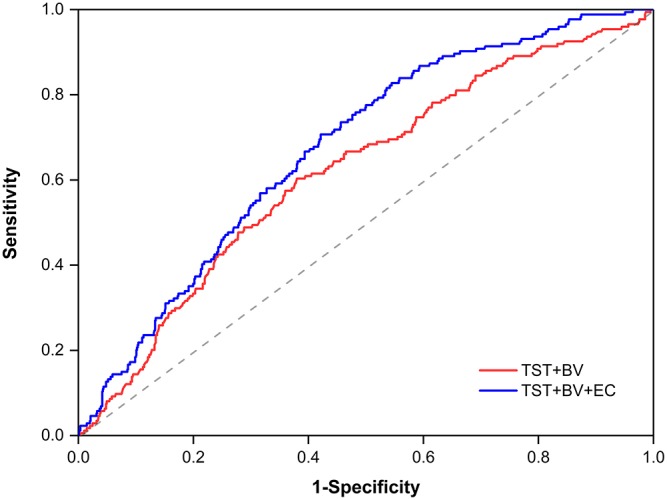
Receiver operating characteristic curves by using a growth definition of >33%, with a binary logistic regression model incorporating BV, TST, and EC (AUC = 0.675) or BV and TTS only (AUC = 0.620). BV, baseline hematoma volume; TST, time from symptom onset to initial computed tomography; EC, eosinophil count; AUC, area under the receiver operating characteristic curve.

The receiver operating characteristic (ROC) analyses demonstrated that eosinophil count had the best discrimination ability for HE irrespective of growth definition, with the highest area under the ROC curve (AUC) of 0.640 (95% CI, 0.595–0.685; *p* < 0.001; Table 8) at the definition of >33% growth; the best predictive cut-off value was 45 cells/μL (sensitivity 69.5%, specificity 58.9%). Additionally, when using the same definition, the predicted model incorporating eosinophil count, baseline hematoma volume (BV), and time from symptom onset to initial CT (TST) achieved a 8.9% improvement in the AUC (AUC = 0.675; 95% CI, 0.635–0.715; *p* < 0.001; Figure 1) over a model based on BV and TST alone (AUC = 0.620; 95% CI, 0.575–0.665; *p* < 0.001). The highest AUC of the combined models was 0.731 (95% CI, 0.686–0.778; *p* < 0.001; sensitivity 85.3%, specificity 51.1%; Table 9), obtained when using the HE definition of >12.5 mL expansion.

**Table 8:** Comparison of AUCs of leukocyte counts in predicting HE by different growth definitions.

	**>6 mL**	**>12.5 mL**	**>33%**	**>6 mL or >33%**	**>12.5 mL or >33%**
WBC count	0.542 (0.498–0.587)	0.541 (0.483–0.599)	0.583 (0.536–0.631)	0.563 (0.520–0.606)	0.575 (0.528–0.622)
*P-*value	0.061	0.15	0.001	0.004	0.001
Neutrophil count	0.572 (0.528–0.617)	0.584 (0.526–0.642)	0.625 (0.579–0.672)	0.599 (0.557–0.641)	0.616 (0.570–0.662)
*P-*value	0.001	0.003	<0.001	<0.001	<0.001
Monocyte count	0.553 (0.508–0.587)	0.578 (0.523–0.634)	0.554 (0.508–0.601)	0.547 (0.504–0.590)	0.552 (0.506–0.598)
*P-*value	0.02	0.006	0.024	0.031	0.027
Eosinophil count	0.591 (0.547–0.636)	0.634 (0.581–0.687)	0.640 (0.595–0.685)	0.604 (0.562–0.647)	0.631 (0.586–0.675)
*P-*value	<0.001	<0.001	<0.001	<0.001	<0.001

*AUC, area under the receiver operating characteristic curve; HE, hematoma expansion; WBC, white blood cell*.

**Table 9:** AUCs in the 2 combined predicted models using various growth definitions of HE.

	**AUC (TST+BT)**	***P-*value**	**AUC (TST+BT+EC)**	***P-*value**	**Improvement**
>6 mL	0.710 (0.672–0.747)	<0.001	0.724 (0.688–0.759)	<0.001	2.0%
>12.5 mL	0.710 (0.662–0.758)	<0.001	0.731 (0.686–0.778)	<0.001	3.0%
>33%	0.620 (0.575–0.665)	<0.001	0.675 (0.635–0.715)	<0.001	8.9%
>6 mL or >33%	0.668 (0.629–0.707)	<0.001	0.690 (0.653–0.727)	<0.001	3.3%
>12.5 mL or >33%	0.642 (0.601–0.684)	<0.001	0.679 (0.639–0.720)	<0.001	5.8%

*AUC, area under the receiver operating characteristic curve; HE, hematoma expansion; TST, time from symptom onset to initial CT; BV, baseline ICH volume; EC, eosinophil count*.

## Discussion

Our study revealed that peripheral counts of WBC, neutrophil, and monocyte on admission were independently associated with the risk of early hematoma enlargement. However, these associations could be affected by HE definitions. Intriguingly, we found a robustly correlation between eosinophil count and increased risk of HE. Furthermore, as compared with other leukocyte types, eosinophil count had the best discrimination ability for HE irrespective of growth definition.

There is no universally agreed definition for HE. A predicted model used an absolute growth definition of 6 mL increase to determine the risk factors associated with HE (19). The relative growth definition of >33% was used by many studies (3, 5, 21), since it could improve the detection rate of HE for ICH that presented with a small volume upon admission. In another prediction model, HE was defined as the combination of 6 mL and 33% increase (18), which was also widely applied in recent investigations (22–24). When using the absolute growth of >12.5 mL, the HE rate we observed was the lowest, consisting with the previous publication (4). In addition to using the growth definition with a specific cut-point, any degree of growth was adopted to define HE as well (17). One drawback was that the observation of HE would be susceptible to measurement errors, especially in patient with a similar follow-up and baseline ICH size. Hence, we did not analyze this definition in our study.

The present study systematically delineated the relationship of leukocyte counts with the most commonly used definitions of HE. Our findings were generally in accordance with the published literature (8), suggesting that the risk of HE is inversely associated with WBC and neutrophil count and directly associated with monocyte count. Interpreting the divergent effect of differential leukocyte counts on the hematoma progression needs to stand on the pathophysiological perspectives. A pronounced inflammation occurs rapidly once the intracranial hematoma is formed, inducing perihematomal leukocytes infiltration within hours (6, 11). In addition to acting on the components within hematoma, exudative leukocytes can interact with platelets and coagulation factors, and promote thrombus formation through shifting the clotting balance (25–27). A procoagulant state caused by the acute inflammation may limit further hematoma enlargement. Neutrophils and monocytes are predominant WBCs, and both enter the brain from the blood-brain barrier after ICH (6, 9). Beyond the bactericidal actions known in the immune response, they have been found to play roles in coagulation pathway. Activated neutrophils can release tissue factor (TF) and downregulate the TF pathway inhibitor in favor of thrombus formation (28, 29). Through interacting with platelets, factor X and XII, neutrophils can enhance thrombin generation and stabilize fibrin (29, 30). On the contrary, the membrane surface of monocyte is shown to be rich in anticoagulant factors such as TF pathway inhibitor, endothelial protein C receptor, and thrombomodulin (26, 31, 32), which may physiologically inhibit thrombus formation and promote fibrinolysis. These preclinical data on the relationship of leukocytes with coagulation components improve the biological rationality of our results.

In clinical studies, the relevance of leukocyte counts to ICH prognosis has attracted mounting attention. Some suggested that admission WBC count was irrelevant to adverse ICH prognosis (13–15, 17). Two multicenter studies reported that higher monocyte count at admission was independently associated with 30-day case fatality, whereas neutrophil and total WBC count were not (15, 17). The researchers conjectured that HE may be the link between monocyte count and poor ICH outcome, and this hypothesis was confirmed by our results. Besides, investigators from the INTERACT2 found no association between elevated WBC count and disability or death at 3 months in patients with acute ICH, after adjusting for baseline clinical and imaging confounders (13). In our study, admission WBC count was inversely associated with the risk of HE, indicating a beneficial role of the acute inflammation in limiting HE, and providing indirect evidence for previous literatures.

Eosinophil is a multifunctional leukocyte that involves in the pathogenesis of diverse inflammatory processes (33), but its specific role in ICH has not yet been elucidated. The mechanism explaining the association of eosinophil count with augmented risk of HE is unclear. Due to the quantitative correlation with total WBC count, one could speculate that our results may only reflect an indirect connection, rather than a causality. Nevertheless, eosinophil count may be a useful biomarker to predict HE. In our study, eosinophil count was robustly correlated with HE irrespective of growth definition, and had the best discrimination ability as compared with other leukocyte types. Baseline ICH volume and onset-to-scan time are strong predictors of HE (2, 34), and are two commonly used variables in the currently available models (18–20). Our results remained significant after adjusting for the two risk factors in the multivariable analyses. Besides, our findings suggest that routine clinical model incorporating eosinophil count could improve the discrimination ability for HE, which might help select more patients with acute ICH for hemostasis treatment in clinical trials.

We did not find an association of HE with WBC and neutrophil count when using the absolute definition of 12.5 mL growth or the combined definition of 12.5 mL with 33% growth. In contrast, a positive association between monocyte count and ICH expansion risk was observed under the definition of >12.5 mL growth only. Although the mechanisms underlying our observations are poorly understood, our findings provide a reasonable explanation for the inconsistent role of monocytes in HE (8, 17). Additionally, studies aimed at identifying risk factors of HE could be based on multiple growth definitions, which may be conducive to capture a comprehensive result. As the divergent effect of neutrophils and monocytes on hematoma progression, future therapeutic strategies should lay particular emphasis on precise regulation of the immune response instead of an extensive anti-inflammatory treatment. Given the intimate relationship between eosinophil count and expansion risk, the role of this leukocyte subtype in the pathophysiology of ICH also merits close attention in basic research. Once the underlying mechanisms are uncovered, this inflammatory marker may provide a novel target for anti-expansion treatment in patients with acute ICH.

This study has several limitations. First, our results are based on a single-center, retrospective analysis. Second, patients who received anticoagulant or antiplatelet treatment before ICH are exclude from this study, thus our results may not apply to these populations. In addition, we perform correlation analyses only upon the most commonly used HE definitions, which may also limit the universality of our results. Third, inflammatory marker such as C-reactive protein, is a predictor of HE (35). We are not able to control for this confounder in our analyses due to lack of emergency laboratory data. Future research needs to clarify whether C-reactive protein has an impact on the relationship between leukocyte counts and HE. Finally, pre-hospital treatment or some specific conditions diagnosed insufficiently might have affected leukocyte counts, although we eliminate them to the best knowledge.

## Conclusions

Among patients with acute spontaneous ICH, admission counts of WBC, neutrophil, and monocyte are independently associated with the risk of HE. However, using different HE definition affects the stability of theses associations. Eosinophil count robustly predicts HE regardless of the growth definition. This laboratory parameter is widely-accessible, and may be a useful biomarker for the risk stratification of early hematoma enlargement in emergency work.

## Data Availability Statement

The datasets generated for this study are available on request to the corresponding author.

## Ethics Statement

This study was carried out in accordance with the recommendations of the Medical Ethics Committee of The First Affiliated Hospital of Wenzhou Medical University, and written informed consent from all subjects was waived due to the retrospective nature of this study. The protocol was approved by the Medical Ethics Committee of The First Affiliated Hospital of Wenzhou Medical University.

## Author Contributions

YY, SH, and XY contributed to the study conception and design. YY acquired the funding. QC, HX, WH, LJ, YX, YL, CZ, and JC collected the patients' data. JL and QC checked the data and performed statistical analyses. QC drafted the article. YY, SH, and JL critically revised it. All authors reviewed the final manuscript and approved it to be submitted.

### Conflict of Interest

The authors declare that the research was conducted in the absence of any commercial or financial relationships that could be construed as a potential conflict of interest.
